# Gestational Medication Use, Birth Conditions, and Early Postnatal Exposures for Childhood Asthma

**DOI:** 10.1155/2012/913426

**Published:** 2011-12-04

**Authors:** Yang-Ching Chen, Ching-Hui Tsai, Yungling Lee

**Affiliations:** ^1^Institute of Epidemiology and Preventive Medicine, College of Public Health, National Taiwan University, Taipei 100, Taiwan; ^2^Department of Family Medicine, Taipei City Hospital, Zhong Xing Branch, Taipei 103, Taiwan

## Abstract

Our aim is to explore (1) whether gestational medication use, mode of delivery, and early postnatal exposure correlate with childhood asthma, (2) the dose responsiveness of such exposure, and (3) their links to early- and late-onset asthma. We conducted a matched case-control study based on the Taiwan Children Health Study, which was a nationwide survey that recruited 12-to-14-year-old school children in 14 communities. 579 mothers of the participants were interviewed by telephone. Exclusive breastfeeding protected children from asthma. Notably, childhood asthma was significantly associated with maternal medication use during pregnancy, vacuum use during vaginal delivery, recurrent respiratory tract infections, hospitalization, main caregiver cared for other children, and early daycare attendance. Exposure to these factors led to dose responsiveness in relationships to asthma. Most of the exposures revealed a greater impact on early-onset asthma, except for vacuum use and daycare attendance.

## 1. Introduction

Asthma is a common childhood disease of complex etiology. Early-life environmental exposures are critical in determining susceptibility to asthma and allergic disease. Large epidemiological studies suggest that the key time period for the development of childhood asthma occurs between conception and 3 years old [1]. Both *in utero* exposure and early-life factors play important roles in the development of airway inflammation and hyper responsiveness [2]. The environmental factors that induce atopy may act together to affect the immune system, which later plays a part in the development of childhood wheezing or asthma.

Several prenatal, perinatal, and early postnatal risk factors have been reported to be related to childhood asthma. In the prenatal aspect, gestational Acetaminophen use might increase the risk of asthma [3]. In the perinatal aspect, some studies have shown that cesarean section causes an increased risk of asthma, but large population studies did not establish a clear degree of consistency [4]. Being born at gestational age of less than 37 weeks and having a low birth weight are well-known risks for asthma development [5]. In the early postnatal risk factors, issues regarding breastfeeding have yielded inconsistent results due to methodological differences and flaws in study design, the immunologic complexity of breast milk, and the possible genetic differences among patients [6]. Even age of daycare attendance might influence the risk of asthma [7]. Recurrent respiratory infections during early childhood also play an important role in asthma incidence [8]. Although several research papers have focused on environmental exposures for childhood asthma, few studies have discussed the influence of the timing of exposure and proved the exposure-asthma relationships in a dose-response manner. On the other hand, based on the background of popular use of herbal medicine during the pregnancy in Taiwan [9], we do not find any study exploring the relationship between gestational herbal medication use, and asthma.

In the present study, we explore in detail the relationships between early-in-life exposure to environmental factors such as gestational medication use, birth conditions, breastfeeding, daycare attendance, and recurrent respiratory tract infections and asthma in Taiwanese children. Our aims were to test (1) whether exposure in the environment to these factors would elevate the risk of asthma, (2) the dose responsiveness of such exposure, and (3) their links to early- and late-onset asthma. We designed a case-control study using data from our previous Taiwan Children Health Study (TCHS) cohort.

## 2. Methods

### 2.1. Subject Selection

We conducted the present study focusing on the TCHS population. Details of the TCHS have already been described [10]. Briefly, the TCHS was a nationwide population-based study that recruited 5,804 seventh, and eighth-grade children from the public schools of 14 Taiwanese communities in 2007. A parent of each child provided written informed consent and completed a self-administered questionnaire. Data from the TCHS was considered baseline data.

A matched sampling design was used to select participants for this nested case-control study. Our study consisted of 4,982 of the 5,804 children, who were nonsmoking children aged 12 to 14 years old at the time of enrollment in the TCHS. Based on 3 age groups, 2 genders, and 14 communities, we then divided asthma- and wheeze-free children into 84 age-, sex-, and community-specific stratas. Controls were randomly selected based on a 1 : 2 principal according to the number of cases in each stratum. Therefore, our case group and the control group were matched by age, sex, and same community. In a structured telephone interview, the biological mothers of the participants were asked to provide details about their children, including demographics, family history of atopic diseases, gestational medication use, feeding practices in infancy, child's age when starting day care attendance, and episodes of respiratory infection events. Children unaccompanied by their biological mothers were excluded from our study population. Three well-trained field workers performed the telephone interviews by using standardized interview skills. All participants provided informed consent. The study protocol was approved by the Institutional Review Board at our university hospital, called the “National Taiwan University Hospital Research Ethics Committee”, and we complied with the principles outlined in the Helsinki Declaration [11].

### 2.2. Exposure Assessment

With regards to prenatal exposures, we focused on detailed medication use status during maternal pregnancy, including different categories of medication and herbal medication as well. Medication tablet use was defined as medications which were prescribed by physicians and was categorized into hypnotics, antisemetics, antibiotics, tocolytic medication, analgesics, and medication for the common cold or gastrointestinal symptoms. General herbal medications for pregnant women in Taiwan, such as Bazhen Tang, Si Wu Tang, Ginseng, Coptis root, and antiabortion herbs (An-Tai-Yin) were recorded. When the mother had a history of taking medication during pregnancy, we also asked about the time, frequency, and duration of the medication use.

 As to perinatal risks, we were concerned about the modes of delivery, gestational age at birth and birth weight of the child. For those from vaginal delivery, we also asked whether vacuum or forceps were used. Preterm delivery was defined as birth before 37 weeks in gestational age, and low birth weight was defined as birth weight lower than 2500 gram. Special peripartum events and congenital defects were also recorded.

For early postnatal exposures, we gathered information about breastfeeding, respiratory tract infections, hospitalization due to respiratory tract infections and whether or not the main caregiver cared for other children in the child's first year of life. We encouraged the mothers to remember the diagnosis of the respiratory tract infections. Choices of respiratory tract infections included common cold, sinusitis, pneumonia, bronchiolitis, croup, acute respiratory distress syndrome, and unknown etiology. We also asked how early the child was sent to a daycare center. Upon determining ever-exposed status, we further inquired about the exposure duration and frequency, such as duration of exclusive breastfeeding, and times of respiratory tract infections and hospitalizations.

### 2.3. Case Definition

We distinguished asthma cases and controls by asking two questions: “Has your child ever experienced difficulty breathing, or have you observed any wheezing or whistling from their chest?” and “Has a doctor ever diagnosed your child as having asthma?” If the answers were “Yes” to both questions, we considered the child as an asthma case. If the answers were “No” to both questions, we considered the child as a control subject. We excluded those with inconsistent data to avoid information bias. Among the original 369 physician diagnosed asthma cases from our cohort, 287 cases were confirmed with “yes” to both questions during our telephone interview. We classified the age of onset as early onset (3 years old and below) and late-onset (after 3 years old) in order to reach similar numbers of participants in each group.

### 2.4. Statistical Analysis

For our matched case-control study design, we used conditional logistic regression to assess the risk of childhood asthma for each individual type of exposure. Odds ratios (ORs) and 95% confidence intervals represented the effects of each risk factor on developing childhood asthma. We further analyzed the different types of asthma by defining the asthma as early onset and late onset. ORs for the association of such exposures with early-onset and late-onset asthma were computed using the conditional likelihood method for multinomial logistic regression models. Chi-squared test of linear trend has been done to present the dose responsiveness. The missing information among these participants was also included in the model by using missing indicators [12]. Besides some a priori confounders based on previous research, we included a covariate if the estimate effects changed by at least 10% in confounder selection. All of the models were adjusted for the parents' level of education, family history of asthma, family history of atopy, mother's age at birth of this child, and *in utero* exposure to maternal smoking. Family history included the past atopy or asthma history of the parents. An independent T test was used to calculate the difference of mother's age at birth of this child between cases and controls. All tests were two-sided at a 5% significance level, and our study participant number was sufficient to reach 80% power. We used SAS version 9.1 (SAS Institute, Cary, NC, USA) software for our statistical analyses.

## 3. Results

Table 1 showed the demographic characteristics for all asthma cases and controls. Ultimately, 579 mothers were recruited in our study. 67.2% of the biological mothers of the 287 asthma cases in TCHS completely responded to our telephone interview. Of the 193 asthma cases, 91 (47.2%) were defined as early-onset and 102 (52.8%) were defined as late-onset asthma. We noticed that our control and original population were of similar frequency in sex, family history of asthma, and atopy (see Table S2 in supplementary material available online at doi:10.1155/2012/913426). The proportions of participants with a positive family history of asthma were 10.8% of asthma cases and 2.1% of the controls (Table 1). Similarly, asthma children had a higher probability of a family history of atopy (45.4%) than the control subjects (27.9%). The average age for the mother at birth of this child was 26.9 in case group and 28.4 in the control group (*P* = 0.01). For asthma children, 2.1% experienced *in utero* exposure to maternal smoking, while only 1.0% of the healthy group had been exposed to maternal smoking during the mother's pregnancy. This case-control study could represent the original cohort as we compared the frequency of control group to the control frequency corrected for sampling of the original population. Besides, our cases and original cases were of similar frequency in sex, family history of asthma, and atopy, and *in utero* maternal smoking (Table  S3).

Exposure to medication or herbal medication during maternal pregnancy was positively associated with childhood asthma (Table 2). 18.9% of mothers among our participants had taken some kinds of medication tablets during their pregnancy. Among those taking medication, nearly two-thirds were medication tablets for the common cold. Any medication tablet use was associated with an increased odds of asthma (OR = 3.73; 95% CI, 2.2–6.33). Dose responsiveness on exposure duration was all significant (*P* for trend <0.05). The odds of asthma increased up to six times comparing to nonexposed group when the exposure occurred during the first trimester (Table 2). Taking medications for the common cold correlated with asthma positively (OR = 4.35; 95% CI, 2.28–8.3). The effect was greater for early-onset asthma as compared to late-onset asthma (OR = 5.31 versus 4.05). 58.9% of herbal medications taken by the mothers during pregnancy belonged to antiabortion herbs (An-Tai-Yin). We did not observe significant relationships between gestational herbal medication use and childhood asthma. However, this exposure correlated with early-onset asthma positively (OR = 3.63; 95%CI, 1.23–10.7) with significant dose responsiveness (*P* for trend <0.05).

Table 3 shows the impact of birth conditions on childhood asthma. Vacuum suction usage during vaginal delivery significantly influences the odds of asthma (OR = 3.68; 95%, 1.34–10.1). This impact was higher for late-onset asthma (OR = 5.16; 95% CI, 1.29–20.6). Additionally, being born before 37 weeks of gestational age was associated with an increased odds of asthma (*P* for trend <0.05), especially the early-onset type.

The influence of early postnatal exposures on childhood asthma is outlined in Table 4. Exclusive breastfeeding showed a decreased odds of asthma (OR = 0.49; 95% CI, 0.31–0.79), especially for early-onset asthma (OR = 0.29; 95%CI, 0.13–0.65). Both recurrent respiratory tract infections (OR = 2.67; 95% CI, 1.78–4.02) and hospitalization in the first year of life (OR = 2.99; 95% CI, 1.58–5.67) were associated with increased odds of asthma, especially for early-onset asthma. Daycare attendance before the child was 3 years old correlated with greater odds of asthma (OR = 2.47; 95% CI, 1.33–4.57). However, this early-life exposure showed a greater influence on late-onset asthma (OR = 4.25; 95% CI, 1.55–11.7). Moreover, we found that the more children the caregiver raised in the first year life, the higher the odds of early-onset asthma (OR = 2.04; 95% CI, 1.13–3.69).

## 4. Discussion

Our findings suggested that the complex etiology of asthma could be traced back from *in utero*, perinatal to early postnatal period. In the present case-control study, we found that childhood asthma was positively related to a series of early-life events, such as *in utero* exposure to maternal medication/herbal medication use, vacuum use and being born before 37 weeks of gestational age during the perinatal period, recurrent respiratory tract infections, hospitalization, and daycare attendance in the early postnatal stage. Exposure to these factors led to dose responsiveness in the risk of asthma. Exclusive breastfeeding was negatively related with childhood asthma. To our knowledge, this is the first study which showed that gestational herbal medication exposures affected childhood asthma positively. Most of the exposures revealed a greater impact on early-onset asthma, except for vacuum use and daycare attendance. These early-life exposures may contribute to the earlier onset of childhood asthma. Several lines of evidence suggest that children who will go on to have more severe and persistent asthma symptoms already have first episode of airway obstruction in their infancy [13]. Hence, in order to prevent those relevant factors of childhood asthma, it would be important to distinguish different associated factors which will influence early- or late-onset of asthma more.

The safety of many common medications used in pregnancy has yet to be confirmed. According to a large prospective cohort study, medication use was reported in 39.2% of pregnancies and herbal medicine use was reported in 0.58% of pregnancies [14]. As compared to studies in Western countries, our prevalence of gestational medication use was lower (18.9%), but use of herbal medications during pregnancy was higher (8.1%). In the present study, we noted that the majority of medication tablets used during pregnancy were cold medications. Most cold medications contain a combination of decongestants, antitussives, expectorants, and mild analgesics which include acetaminophen and nonsteroidal anti-inflammatory drugs (NSAIDs) [15]. Many studies have shown that prenatal exposure to acetaminophen may increase the risk of asthma, which was compatible with our findings with regards to cold medication use (Table 2). The possible mechanism of the association between acetaminophen and asthma has been hypothesized to involve the glutathione pathway which is downregulated following acetaminophen exposure [16].

The use of herbal medicine during the pregnancy period is common in Taiwan [9]. We surveyed some of the commonly used herbal medications in Taiwan, such as Bazhen Tang, Si Wu Tang, Ginseng, Coptis root, antiabortion herbs (An-Tai-Yin). Bazhen Tang and Si Wu Tang are used for better growth in fetus weight. Ginseng is used to avoid abortion in the early stage of pregnancy. Coptis root is taken to solve the skin problems of pregnant women and to enhance the beauty of the skin of infants. An-Tai-Yin is used in pregnant women under risk of abortion to avoid abortion of fetus. We found that more than half of the herbal medications taken by the mothers during pregnancy belonged to antiabortion herbs (An-Tai-Yin). In a previous Taiwanese study, it was reported that An-Tai-Yin use during the first trimester was associated with an increased risk of congenital malformations of the musculoskeletal and connective tissues [17]. Until now, no study has been conducted on the relationships between gestational herbal medication use and asthma. In our study, we found that gestational herbal medication use was significantly associated with increased odds of early-onset asthma. The more frequent the herbal medication use by mothers, the higher the risk of asthma for their children (Table 2). Because of the incompletely assessed safety and efficacy of herbal medications, avoidance of herbal medicine during pregnancy should be recommended by obstetricians [18].

Reports on the relationships between cesarean section (CS) and childhood asthma are not consistent [4, 19]. “Hygiene hypothesis” suggested that the sterile infant is colonized by bacteria from the hospital environment and skin, not by maternal bacteria from the birth canal and perineum. Therefore, the initial “wrong” microbes can have long-term adverse effects on triggering atopic diseases [4]. Another possible mechanism is that CS is associated with increased risk of newborn respiratory distress syndrome, which might predispose to childhood asthma. Contrary to other studies [20], our result did not reveal a significant relationship between CS and childhood asthma. The results of this study were not consistent with the “hygiene hypothesis” and suggested that there might be a complex interaction between host and microbial agents with heterogeneous effects on the development of asthma [19].

In our data, vacuum-assisted delivery was associated with childhood asthma (Table 3), which was consistent with many previous studies [21]. Vacuum delivery is commonly indicated for prolong labor, maternal exhaustion, or emergencies at the delivery process. Maternal stress is related to excessive cortisol secretion, which further affects the child's immune system and increases the child's susceptibility to allergic disease [22]. In certain emergent circumstances, vacuum-assisted suction would be used in removing meconium aspiration, which could cause damage in lung of an infant. This pathological damage might lead the child to be more susceptible to having asthma in the future [23]. On the other hand, we found an inverse association between gestational age and childhood asthma, especially early-onset asthma. Our result was consistent with one recent meta-analysis based on 19 studies showing that children born prematurely would have an approximately 7% higher risk of asthma as compared with full-term children [5]. Another large population-based study suggested that this association was not solely due to prematurity increasing the risk of respiratory tract infection [24]. The exact mechanisms behind the links between preterm delivery and the development of childhood asthma need further investigation.

We found that exclusive breastfeeding prevents children from having asthma, especially for early-onset asthma (Table 4). The mechanisms for the effect of breastfeeding to protect infants from atopic diseases are quite complex. One is that soluble TGF-*β*, which is predominant in human breast milk, increases the ability of the infant to produce IgA against allergens. Soluble CD 14 in breast milk is thought to be the induction of a Th1 response to bacteria [25]. Another possible mechanism is related to childhood exposure to microbes. Breast milk may provide antiviral antibodies and other factors that reduce the incidence of these infections and subsequent wheezing. In a multicountry meta-analysis, breastfeeding was found to protect against only nonatopic wheezing in nonaffluent countries with OR of 0.69 (95% CI, 0.53–0.90) [26]. However, in another cluster randomized trial, results did not support the protective effect of prolonged and exclusive breastfeeding on childhood asthma [27]. Although our retrospective study design is prone to suffer from recall bias, it has been proven that maternal recall is valid and reliable in estimating breastfeeding initiation and duration [28]. Further extensive studies on the differences of mechanisms between exclusive and nonexclusive breastfeeding in relation to childhood asthma are warranted.

Exposures to recurrent respiratory tract infections and hospitalization in the first year of life were associated with increased odds of early-onset asthma. (Table 4) Early daycare would result in more respiratory infections early in life [7]. Infections with respiratory syncytial virus (RSV), parainfluenza, influenza, and rhinovirus are closely associated with the development of wheezing and pulmonary inflammation [29]. RSV-associated respiratory tract infections represented the most common cause of hospitalization at early infancy. In fact, numerous studies reported associations between RSV bronchiolitis in early childhood and recurrent wheezing and asthma in later childhood [30]. Our findings were consistent with these studies, but disputed the “hygiene hypothesis”, suggesting the protective role of microbial infections during early childhood with regards to the development of asthma. Epidemiologic studies have challenged the “hygiene hypothesis” as being oversimplified [31]. One recent in vivo study suggested that innate immune stimulation via microbes could cause allergen sensitization in a dose- and time-dependent manner [32]. There might be complicated interactions between host and microbial agents in asthma development.

Recall bias is the major limitation in any case-control design. The mothers of children in the present study were informed that the interview was about the children's health problems, instead of explicitly mentioning asthma initially. Identification of asthma was performed at the end of the interview to reduce the possibility of recall bias. Distant recall of prenatal and perinatal factors reported by biological mothers has been considered a reliable approach in previous studies [33–35]. Githens et al. found 102 usable and reliable responses from mothers concerning gestational, perinatal and early-postnatal factors with a recall period of four to six years [35]. Yawn et al. also reported a high degree of agreement between medical records and maternal report for perinatal events such as cesarean section, preexisting diabetes, and smoking in a 10 to 15 years distant recall [33]. Moreover, in Buka et al.'s retrospective survey, women can accurately recall perinatal events such as CS, birth weight, and length of gestation even after 22 years [34]. Furthermore, early-life environmental exposures in this study were not all well-known risk factors for childhood asthma, such as gestational medication use, herbal medication use, and vacuum delivery. The probability of a mother mistakenly recalling the early-life events during her pregnancy such as gestational medication use and her child's life should be comparable in both case and control groups, and a misclassification is likely to be nondifferential [36]. Moreover, significant dose responsiveness in many early-life exposures confirmed the importance of these risk factors. We believe that it is unlikely that a mother would have recall bias about the breastfeeding, hospitalization, and daycare attendance of her child [28, 37]. Given the rise in telephone scams in Taiwan today, the more educated mother in the healthy group tended to decline any telephone interviews from people they do not already personally know. Selection bias may exist in telephone interview process because less educated mothers in the control groups may be more willing to be interviewed. On the contrary, well-educated mothers of asthmatic children would be more willing to answer our 30 minutes telephone interview, because they cared about their child's health more. To compensate for this, we adjusted the level of education of the parents in our statistical models. On the other hand, we noticed that those mothers in our control group were of lower *in utero* smoking prevalence. This is because mothers who are willing to answer our telephone interview were those who cared about their children's health more. Therefore, *in utero* maternal smoking was chosen was a confounder to be adjusted in our statistical model.

## 5. Conclusions

Our data disclosed that gestational medication use, vacuum delivery, recurrent respiratory tract infections, hospitalization, and daycare attendance in early postnatal stage were associated with higher odds of childhood asthma. Exclusive breastfeeding protects children from asthma. Most of the exposures revealed a greater impact on early-onset asthma, except for vacuum use and daycare attendance. We recommend that mothers should be encouraged to breastfeed, to avoid any unnecessary medication during their pregnancy, and to protect children from harmful exposures in their early childhood.

## Supplementary Material

Below we listed the questions we used to get the environmental exposures in the nested case control study (Table S1). In addition, we have demonstrated the representativeness of our cases/controls as compared to the original cohort population in Table S2-S3.

## Figures and Tables

**Table 1:** Demographic characteristics of the study population.

	Case		Control		Control frequency corrected for sampling	
	(*N* = 193)		(*N* = 386)		(*N* = 4312)	
Characteristics	*N*	%	*N*	%	*N* ^‡^	%
Sex						
Girls	84	43.5	168	43.5	1876	43.5
Boys	109	56.5	218	56.5	2436	56.5
Parental education, yr^†^						
≦12	116	61.7	264	69.1	2603	60.4
13~15	41	21.8	69	18.1	888	20.6
≧16	31	16.5	49	12.8	821	19.0
Family history of asthma^†^						
No	165	89.2	372	97.9	4212	97.7
Yes	20	10.8	8	2.1	100	2.3
Family history of atopy^∗†^						
No	101	54.6	274	72.1	3252	75.4
Yes	84	45.4	106	27.9	1060	24.6
*In utero* maternal smoking^†^						
No	189	97.9	382	99.0	4162	96.5
Yes	4	2.1	4	1.0	150	3.5

*Atopy is defined as allergic rhinitis or atopic eczema.

^†^Number of subjects does not add up to total N because of missing data.

^‡^Predicted number of controls in the TCHS cohort based on the sampling.

**Table 2:** Risks for childhood asthma in relation to gestational medication use.

	Controls	Ever asthma			Early-onset asthma†			Late-onset asthma^‡^		
Gestational medication category	*N* (%)^§^	*N* ^§^	OR	95%CI	*N* ^§^	OR	95%CI	*N* ^§^	OR	95%CI
Any medication tablet^¶^										
No	350(90.7)	137	1.00		71	1.00		66	1.00	
Yes	36(9.3)	56	3.73	(2.2,6.33)**	20	3.64	(1.54,8.64)*	36	3.90	(1.96,7.75)**
Exposure time										
2,3 trimester	19(4.9)	20	1.80	(0.86,3.79)	7	3.37	(0.83, 13.63)	13	1.46	(0.58, 3.70)
1st trimester	17(4.4)	33	6.03	(2.91,12.5)**	12	3.32	(1.17, 9.43)	21	10.20	(3.34, 31.17)**
Exposure duration										
0–6 days	28(7.3)	43	3.46	(1.94,6.17)**	15	3.41	(1.31, 8.83)*	28	3.55	(1.69, 7.45)**
≧1 week	8(2.1)	13	4.86	(1.77, 13.37)*	5	4.57	(0.89, 23.56)	8	5.70	(1.43, 22.77)*
*P* for trend			<0.001			0.004			<0.001	
Medications for common cold										
No	351(93.6)	146	1.00		75	1.00		71	1.00	
Yes	24(6.4)	41	4.35	(2.28,8.3)**	13	5.31	(1.63,17.3)*	28	4.05	(1.85,8.87)**
Exposure time										
2,3 trimester	15(4.0)	17	2.19	(0.97,4.94)	6	4.76	(0.88, 25.90)	11	1.82	(0.67, 4.92)
1st trimester	9(2.4)	21	8.67	(3.10,24.23)**	6	4.49	(0.82, 24.48)	15	10.20	(2.78, 37.50)**
Exposure duration										
0–3 days	16(4.3)	23	3.19	(1.50, 6.79)*	8	4.50	(1.19,16.97)*	15	2.77	(1.08, 7.14)*
≧4 days	8(2.1)	18	7.83	(2.70,22.72)**	5	8.84	(0.83, 93.83)	13	7.17	(2.16, 23.75)*
*P* for trend			<0.001			0.008			<0.001	
Herbal medication										
No	361(93.5)	171	1.00		80	1.00		91	1.00	
Yes	25 (6.5)	22	1.59	(0.83,3.05)	11	3.63	(1.23,10.7)*	11	1.07	(0.44,2.61)
Exposure time										
2,3 trimester	8 (2.3)	8	1.41	(0.48, 4.09)	6	5.17	(0.89, 31.18)	2	0.53	(0.09, 2.95)
1st trimester	15 (4.2)	14	1.94	(0.85, 4.41)	5	2.99	(0.71, 12.54)	9	1.59	(0.57, 4.45)
Exposure duration										
<6 days	10 (2.7)	11	1.63	(0.63, 4.23)	7	6.83	(1.22, 38.13)	4	0.65	(0.17, 2.50)
≧1 week	14 (3.8)	11	1.77	(0.73, 4.28)	4	2.33	(0.58, 9.42)	7	1.96	(0.58, 6.59)
*P* for trend			0.12			0.05			0.46	
Exposure frequency										
<3 times/week	19 (5.1)	11	0.82	(0.35,1.94)	6	2.85	(0.68, 12.02)	5	0.41	(0.12, 1.41)
≧4 times/week	5 (1.4)	10	6.64	(1.89,23.32)*	4	4.71	(0.88,25.37)	6	16.70	(1.71, 162.76)
*P* for trend			0.02			0.02			0.14	

Models are adjusted for parental education, mother's age at birth of her child, parental history of asthma, parental history of atopy, and *in utero* maternal smoking.

*P < 0.05; **P < 0.001.

^§^Number of subjects does not add up to total *n* because of missing data.

^†^Early onset: asthma diagnosed ≦3 yr of age.

^‡^Late onset: asthma diagnosed >3 yr of age.

^¶^Any medication: including any medication tablet that physicians prescribed.

**Table 3:** Risks for childhood asthma in relation to delivery modes and birth conditions.

	Controls	Ever asthma			Early-onset asthma^†^			Late-onset asthma^‡^		
	*N* (%)^§^	*N* ^§^	OR	95%CI	*N*	OR	95%CI	*N* ^§^	OR	95%CI
Modes of delivery										
Vaginal	255 (66.2)	128	1.00		61	1.00		67	1.00	
Scheduled CS	58(15.1)	25	1.04	(0.59, 1.83)	13	1.19	(0.49, 2.88)	12	0.97	(0.45, 2.09)
Emergent CS	72(18.7)	38	1.13	(0.70, 1.83)	16	0.76	(0.37, 1.57)	22	1.63	(0.81, 3.28)
Forceps usage during vaginal delivery										
No	250(98.0)	118	1.00		59	1.00		59	1.00	
Yes	5(2.0)	9	2.12	(0.57, 7.91)	2	0.41	(0.04, 3.95)	7	5.10	(0.84, 30.93)
Vacuum usage during vaginal delivery										
No	243(95.3)	103	1.00		51	1.00		52	1.00	
Yes	12(4.7)	24	3.68	(1.34, 10.1)*	10	2.01	(0.47, 8.63)	14	5.16	(1.29, 20.60)*
Low birth weight										
No	338(90.4)	158	1.00		78	1.00		80	1.00	
Yes	36(9.6)	31	1.73	(0.96, 3.1)	13	1.99	(0.77, 5.13)	18	1.76	(0.81, 3.8)
2000–2500 gm	25(6.7)	22	1.70	(0.86, 3.35)	9	1.61	(0.56, 4.62)	13	1.60	(0.72, 3.55)
< 2000 gm	11(2.9)	9	1.79	(0.66, 4.87)	4	4.18	(0.65, 26.77)	5	5.69	(0.49, 65.67)
*P* for trend			0.08			0.10			0.09	
Preterm delivery^¶^										
No	356(92.2)	169	1.00		80	1.00		89	1.00	
Yes	30(7.8)	24	1.83	(0.98, 3.41)	11	2.47	(0.94, 6.50)	13	1.4	(0.63, 3.27)
34–36 wk	22(7.2)	16	1.74	(0.81, 3.74)	8	2.51	(0.82, 7.72)	8	1.16	(0.40, 3.36)
< 34 wk	2(0.7)	5	4.31	(0.81, 2.88)	3	4.80	(0.44, 52.14)	2	4.48	(0.39, 51.97)
*P* for trend			0.03			0.04			0.32	

Models are adjusted for parental education, mother's age at birth of her child, parental history of asthma, parental history of atopy, and *in utero* maternal smoking.

*P < 0.05.

^§^Number of subjects does not add up to total *n* because of missing data.

^†^Early onset: asthma diagnosed ≦3 yr of age.

^‡^Late onset: asthma diagnosed >3 yr of age.

^¶^Preterm delivery was defined as children born at gestational age less than 37 weeks.

**Table 4:** Risks for childhood asthma in relation to early postnatal exposures.

	Controls	Ever asthma			Early-onset asthma^†^			Late-onset asthma^‡^		
Early postnatal exposures	*N* (%)^§^	*N*	OR	95%CI	*N*	OR	95%CI	*N*	OR	95%CI
Exclusive breastfeeding										
No	268 (69.4)	159	1.00		77	1.00		82	1.00	
Yes	118(30.6)	33	0.49	(0.31, 0.79)*	13	0.29	(0.13, 0.65)*	20	0.67	(0.37, 1.2)
Exclusive BF < 1 month	55 (14.3)	14	0.41	(0.21, 0.77)*	7	0.28	(0.10, 0.76)*	7	0.49	(0.20, 1.17)
Exclusive BF≧1 month	63 (16.3)	19	0.60	(0.32, 1.11)	6	0.30	(0.09, 0.95)*	13	0.86	(0.41, 1.80)
*P* for trend			0.01			0.007			0.35	
Recurrent respiratory infections^¶^										
No	248(64.6)	79	1.00		33	1.00		46	1.00	
Yes	136(35.4)	111	2.67	(1.78, 4.02)**	56	4.07	(2.01, 8.27)**	55	2.26	(1.35, 3.81)*
Frequency										
3–6 times/year	91 (23.7)	46	1.55	(0.95, 2.53)	18	1.64	(0.68, 3.96)	28	1.64	(0.90, 3.02)
≧7 times/year	45 (11.7)	65	5.36	(3.12, 9.22)**	38	9.42	(3.84, 23.12)**	27	3.93	(1.86, 8.32)**
*P* for trend			<0.001			<0.001			<0.001	
Hospitalization within first year of age										
No	362(94.3)	163	1.00		73	1.00		90	1.00	
Yes	22(5.7)	30	2.99	(1.58, 5.67)**	18	4.04	(1.64, 9.96)*	12	2.24	(0.86, 5.83)
Frequency										
1 times	15(4.3)	23	3.56	(1.68, 7.51)*	12	3.53	(1.22, 10.24)*	11	3.40	(1.14, 10.10)*
≧2 times	5(1.4)	7	2.82	(0.84, 9.53)	6	12.21	(1.30, 115.07)*	1	0.62	(0.07, 5.80)
*P* for trend			0.001			0.002			0.29	
Daycare attendance within three years of age										
No	354 (91.7)	161	1.00		74	1.00		87	1.00	
Yes	32 (8.3)	30	2.47	(1.33, 4.57)*	17	1.77	(0.76, 4.12)	13	4.25	(1.55, 11.7)*
2-3 years old	17 (4.4)	18	3.12	(1.41, 6.93)*	9	1.70	(0.57, 5.03)	9	8.26	(1.94, 35.27)*
< 2 years old	15 (3.9)	12	1.85	(0.78, 4.40)	8	1.85	(0.59, 5.75)	4	1.90	(0.43, 8.34)
*P* for trend			0.02			0.20			0.03	
Main caregiver cared for other children										
No	231(59.8)	96	1.00		43	1.00		53	1.00	
Yes	155(40.2)	97	1.51	(1.02, 2.23)*	48	2.04	(1.13, 3.69)*	49	1.13	(0.65, 1.97)
Number of children^||^										
1	108(27.9)	67	1.42	(0.92, 2.20)	33	2.02	(1.04, 3.92)*	34	1.01	(0.55, 1.86)
≧2	47(12.1)	30	1.73	(0.97, 3.08)	15	2.10	(0.87, 5.06)	15	1.48	(0.67, 3.29)
*P* for trend			0.04			0.03			0.42	

Models are adjusted for parental education, mother's age at birth of her child, parental history of asthma, parental history of atopy, *in utero* maternal smoking.

*P < 0.05; **P < 0.001.

^§^Number of subjects does not add up to total *n* because of missing data.

^†^Early onset: asthma diagnosed ≦3 yr of age.

^‡^Late onset: asthma diagnosed >3 yr of age.

^¶^Recurrent respiratory infections were defined as more than three times of respiratory infections in the first year of life.

^||^The number of other children the main caregiver cared for in the first year of life.

BF: breastfeeding.

## Abbreviations

CS: Cesarean section
RSV: Respiratory syncytial virus
TCHS: Taiwan Children Health Study.
