# Supplementary material for: Gestational Medication Use, Birth Conditions, and Early Postnatal Exposures for Childhood Asthma
Source: Clin Dev Immunol. 2011 Dec 4;2012:913426. doi: 10.1155/2012/913426 (PMC3235498; doi:10.1155/2012/913426)
Supplement: Supplementary file 1 — Below we listed the questions we used to get the environmental exposures in the nested case control study (Table S1). In addition, we have demonstrated the representativeness of our cases/controls as compared to the original cohort population in Table S2-S3. [file 913426.f1.pdf]

Table S1. Environmental questions used in the nested case control study

1. During your pregnancy, did you take any medication?
   (1) None; (2) Yes

   If yes, then:
   (1) What kind of medication did you take?
   (A) Anti-pyretics (B) Hypnotics
   (C) Gastro-intestinal medication (D) Common cold medication
   (E) Tocolysis (F) Anti-emetics
   (G) Antibiotics (H) Others

   (2) When did you start to take medication during your pregnancy?
   (A) 1st trimester (pregnancy for 1-4 months) (B) 2nd trimester (pregnancy for 5-7 months)
   (C) 3rd trimester (pregnancy for 8-10 months)

   (3) For how long did you take the medication (the earliest kind)?
   (A) 0-3 days (B) 4-7 days
   (C) 1-2 weeks (D) 2-4 weeks
   (D) More than 1 month

2. During your pregnancy, did you take any Chinese herbal medication?
   (2) None; (2) Yes

   If yes, then:
   (1) What kind of Chinese herbal medication did you take?
   (A) Bazhen Tang (B) Si Wu Tang
   (C) Ginseng (D) Coptis root
   (E) Anti-abortion herbs (An-Tai-Yin) (F) Unknown
   (G) Others

   (2) When did you start to take Chinese herbal medication during your pregnancy?
   (A) 1st trimester (pregnancy for 1-4 months) (B) 2nd trimester (pregnancy for 5-7 months)
   (C) 3rd trimester (pregnancy for 8-10

months)

(3) What is the frequency of taking Chinese herbal medication during your pregnancy?
(A) <1 time/month (B) At least 1 time/month
(C) 1-3 times/week (D) 4-6 times/week
(E)At least 1 time daily

(4) What is the duration of taking Chinese herbal medication during your pregnancy?
(A) <3 days (B)3-7 days
(C) 1-2 weeks (D)2-4 weeks
(E)more than 1 month

3. What is the mode of delivery when you give birth to this child
(1) Vaginal delivery; (2) Cesarean section.

4. During the process of delivery, did your doctor use forceps during vaginal delivery?
(1) None; (2) Yes

5. What was the birth weight of your child? _________gram
6. What was the gestational age while you give birth to this child? _________weeks
7. Have you exclusively breastfed your child?
(1) None; (2) Yes

If yes, then for how long?
(1) Less than one month (2) 1-2 month
(3) 2-4 months (4) 4-6 months
(5) 6-8 months (6) 8-10 months
(7) 10-12 months (8) More than 1 year

8. How many times did your child have respiratory tract infection before he (she) was 1 year old ?
(1) None; (2) 1-2 times; (3) 3-4 times; (4) 5-6 times; (5) more than 6 times.

9. Did your child hospitalize due to respiratory tract infection before he (she) was 1 year old ?
(1) None; (2) Yes

10. Did your child attend daycare center before he (she) enter elementary school?
    (1) None;   (2) Yes

    If yes, when did your child attend daycare center?

(A) 0-4 months	(B) 4-6 months
(C) 6-12 months	(D) 1-2 years-old
(E) 2-3 years-old	(F) 3-4 years-old
(G) 4-7 years-old	(H) More than 7 years-old

11. How many children other than your child did the main caregiver care before your child was 1 year old ?

(1) None	(2) 1 child
(3) 2 children	(4) 3 children
(5) More than 3 children	

Table S2. The representativeness of our control groups as compared to original control population.

	**Control**		**Control frequency corrected for sampling**		**Difference between control and original population**
	**(N=386)**		**(N=4312)**		
**Characteristics**	N	%	N‡	%	P value
**Sex**					0.99
**Girls**	168	43.5	1876	43.5	
**Boys**	218	56.5	2436	56.5	
**Parental education, yr†**					0.001
**≦12**	264	69.1	2603	60.4	
**13~15**	69	18.1	888	20.6	
**≧16**	49	12.8	821	19.0	
**Family history of asthma†**					0.79
**No**	372	97.9	4212	97.7	
**Yes**	8	2.1	100	2.3	
**Family history of atopy*†**					0.15
**No**	274	72.1	3252	75.4	
**Yes**	106	27.9	1060	24.6	
***In utero* maternal smoking †**					0.01
**No**	382	99.0	4162	96.5	
**Yes**	4	1.0	150	3.5	

*Atopy is defined as allergic rhinitis or atopic eczema.

†Number of subjects does not add up to total N because of missing data.

Table S3. The representativeness of our cases as compared to original cases in baseline cohort.

	Case		Original cases		Difference between control and original population
	(N=193)		(N=287)		
**Characteristics**	N	%	N	%	P value
**Sex**					0.59
**Girls**	84	43.5	132	45.9	
**Boys**	109	56.5	155	54.1	
**Parental education, yr†**					0.01
**≦12**	116	61.7	174	60.6	
**13~15**	41	21.8	93	32.5	
**≧16**	31	16.5	20	6.9	
**Family history of asthma†**					0.37
**No**	165	89.2	263	91.8	
**Yes**	20	10.8	24	8.2	
**Family history of atopy*†**					0.25
**No**	101	54.6	172	59.9	
**Yes**	84	45.4	115	40.1	
***In utero* maternal smoking †**					0.06
**No**	189	97.9	271	94.5	
**Yes**	4	2.1	16	5.5	

*Atopy is defined as allergic rhinitis or atopic eczema.

†Number of subjects does not add up to total N because of missing data.
